# Dengue Virus Infection and Associated Risk Factors in Africa: A Systematic Review and Meta-Analysis

**DOI:** 10.3390/v13040536

**Published:** 2021-03-24

**Authors:** Gaspary O. Mwanyika, Leonard E. G. Mboera, Sima Rugarabamu, Baraka Ngingo, Calvin Sindato, Julius J. Lutwama, Janusz T. Paweska, Gerald Misinzo

**Affiliations:** 1 SACIDS Africa Centre of Excellence for Infectious Diseases, Sokoine University of Agriculture, P.O. Box 3297 Morogoro, Tanzania; gaspary.mwanyika@sacids.org (G.O.M.); leonard.mboera@sacids.org (L.E.G.M.); sima.rugarabamu@sacids.org (S.R.); ngingobaraka@gmail.com (B.N.); calvin.sindato@sacids.org (C.S.); 2 Department of Veterinary Microbiology, Parasitology and Biotechnology, Sokoine University of Agriculture, P.O. Box 3015 Morogoro, Tanzania; 3 Department of Health Science and Technology, Mbeya University of Science and Technology, P.O. Box 131 Mbeya, Tanzania; 4 Department of Microbiology and Immunology, Muhimbili University of Health and Allied Sciences, P.O. Box 65595 Dar es Salaam, Tanzania; 5 Biology Department, St. John’s University of Tanzania, P.O. Box 47 Dodoma, Tanzania; 6 Tabora Research Centre, National Institute for Medical Research, P.O. Box 482 Tabora, Tanzania; 7 Department of Arbovirology, Emerging and Re-emerging Infectious Diseases, Uganda Virus Research Institute, P.O. Box 49 Entebbe, Uganda; jjlutwama03@yahoo.com; 8 National Health Laboratory Service, National Institute for Communicable Diseases, Sandringham, 2192 Johannesburg, South Africa; janusz@gmail.com

**Keywords:** dengue, prevalence, risk factors, Africa

## Abstract

Dengue contributes a significant burden on global public health and economies. In Africa, the burden of dengue virus (DENV) infection is not well described. This review was undertaken to determine the prevalence of dengue and associated risk factors. A literature search was done on PubMed/MEDLINE, Scopus, Embase, and Google Scholar databases to identify articles published between 1960 and 2020. Meta-analysis was performed using a random-effect model at a 95% confidence interval, followed by subgroup meta-analysis to determine the overall prevalence. Between 1960 and 2020, 45 outbreaks were identified, of which 17 and 16 occurred in East and West Africa, respectively. Dengue virus serotype 1 (DENV-1) and DENV-2 were the dominant serotypes contributing to 60% of the epidemics. Of 2211 cases reported between 2009 and 2020; 1954 (88.4%) were reported during outbreaks. Overall, the prevalence of dengue was 29% (95% CI: 20–39%) and 3% (95% CI: 1–5%) during the outbreak and non-outbreak periods, respectively. Old age (6/21 studies), lack of mosquito control (6/21), urban residence (4/21), climate change (3/21), and recent history of travel (3/21) were the leading risk factors. This review reports a high burden of dengue and increased risk of severe disease in Africa. Our findings provide useful information for clinical practice and health policy decisions to implement effective interventions.

## 1. Introduction

Dengue is an important arboviral disease, with the highest incidence in tropical and subtropical regions, with a potential to spread into other geographical areas. In the past four decades, dengue has caused a significant impact on human health and national economies [1,2]. Approximately 390 million people are infected with dengue virus (DENV) annually. Of these, 96 million develop clinical manifestations that lead to 500,000 hospitalizations and 25,000 deaths, annually [3]. Dengue is caused by an RNA virus of the family *Flaviviridae* that is transmitted to humans through a bite of infected Aedes mosquitoes. Dengue virus exists in four genetically related but antigenically distinct serotypes (DENV-1–4), each with the ability to cause self-limiting fevers to fatal conditions such as dengue hemorrhagic fever (DHF) and dengue shock syndrome (DSS). Although dengue infection confers lifelong immunity after primary infection by one serotype, secondary infection by heterologous serotypes or virulent strains increases the risk of severe disease [4]. During the 19th century, dengue epidemics in Africa were initially reported in the Zanzibar Islands in 1823 and 1870, Burkina Faso in 1925, and South Africa between 1926 and 1927 [5]. In the 1960s, laboratory-confirmed outbreaks started being reported in many other African countries [5]. All four serotypes (DENV-1–4) have been reported in the continent, with DENV-1 and DENV-2 being reported most frequently [6,7]. Despite increasing reports of dengue in Africa, its burden in different epidemiological contexts is not well described. This could be due to inadequate laboratory capacity to differentiate dengue from other febrile illnesses, such as malaria, chikungunya, Zika, yellow fever, typhoid fever, and leptospirosis, that share a similar clinical presentation and geographical distribution [8,9,10]. The objective of this systematic review and meta-analysis was to analyze the prevalence of dengue infection and associated risk factors in Africa.

## 2. Materials and Methods

### 2.1. Search Strategy and Selection Criteria

This review was performed according to Preferred Reporting Items for Systematic Reviews and Meta-analyses (PRISMA) guidelines [11], and the protocol registered in PROSPERO (CDR420202105579). PubMed/MEDLINE, Scopus, Embase, and Google Scholar databases were searched for articles for a period of three months from October to December 2020. Additional literature was searched from African Journals Online, World Health Organization, and Program for Monitoring Emerging Diseases (ProMED) databases. The key search terms were: (Dengue) AND (Africa = list of countries) AND (“Outbreak” OR “Prevalence” OR “Co-morbidities” OR “Risk factors”) (Appendix A). The primary studies describing outbreak incidence, dengue prevalence based on ribonucleic acid (RNA) and non-structural protein 1 (NS1) antigen, co-morbidities, and potential risk factors in the African continent published between 1960 and 2020 were considered for review. We excluded studies with abstracts only, dengue cases in studies not involving human subjects, articles in languages other than English, review papers, and studies with incomplete data. Dengue infection was defined as febrile illness presenting with fever and at least two of the following clinical manifestations: headache, retro-orbital pain, myalgia, arthralgia, rash, hemorrhagic manifestations, and leukopenia confirmed by laboratory criteria through the detection of DENV RNA using reverse transcription polymerase chain reaction (RT-PCR) or NS1 antigens using enzyme-linked immunosorbent assay (ELISA) and/or rapid tests.

### 2.2. Data Extraction and Management

Records on the authors, geographical origin, setting (hospital *versus* community), study design, number of dengue cases, total participants tested and epidemiological context (outbreak *versus* non-outbreak), detected DENV serotypes, co-morbidities, and potential risk factors of dengue infection were extracted into a Microsoft Excel spreadsheet (Excel 2019, Microsoft Corp., Redmond, WA, USA). The duplicates were removed using Rayyan web application software for systematic review [12].

### 2.3. Quality Assessment

The methodological quality of selected prevalence studies was evaluated by two reviewers using a quality assessment checklist adapted from Hoy and others [13]. Risk of bias was assessed using nine domains: target population, sampling frame, sample selection method, likelihood of non-response bias, data source, case definition, study instrument that measured the parameter of interest, mode of data collection, and numerator and denominator of the parameter of interest. The risk of bias levels was low (score = 0) or high (score = 1), and the overall risk of bias was defined as low (score 0–3), moderate (4–6), and high (7–9) (Table S1). Any discrepancy was resolved through discussion with a third reviewer.

### 2.4. Statistical Analysis

The extracted data were pooled using MetaXL version 5.3 software (EpiGear International Pty Ltd., Queensland, QLD, Australia). A random effect model was used to estimate the overall prevalence and 95% confidence intervals (CI), and results were presented in forest plots. The percentage of heterogeneity between studies was quantified using I^2^ and chi-square tests, and I^2^ ≥ 50% was considered significant. Sensitivity analysis to test the effect of each study on summary prevalence, by excluding each study step by step, was used to evaluate the robustness of overall prevalence. A funnel plot and Egger’s regression test were used to detect publication bias. All results with *p*-values < 0.05 were considered statistically significant. Descriptive statistics, narrative synthesis, and relevant figures were used to summarize the information where statistical pooling was not possible.

## 3. Results

### 3.1. Search Results and Characteristics of Selected Studies

**Figure 1:**
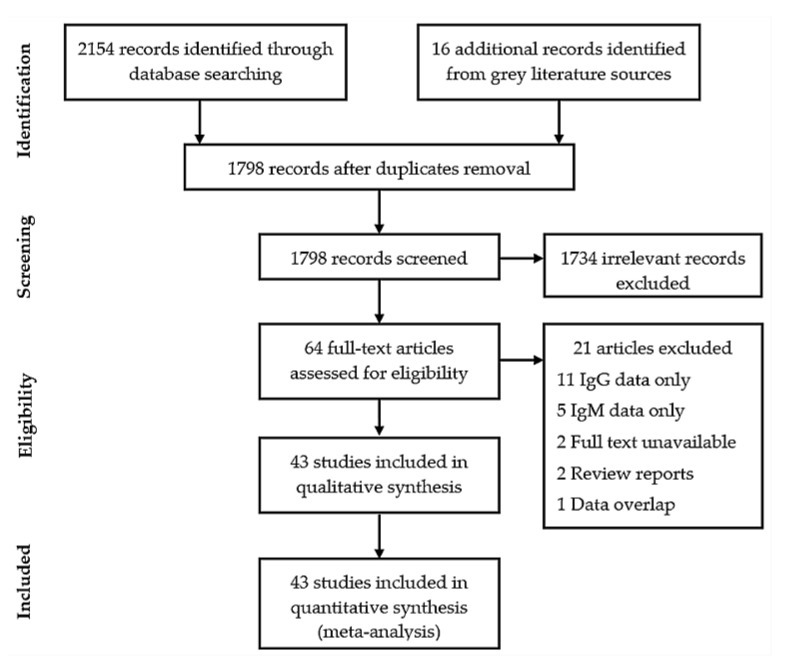
PRISMA flow chart illustrating literature selection and inclusion process.

A total of 2170 records were retrieved from database searches. After duplicates removal and screening, 43 studies were finally included in the review (Figure 1). The methodological quality of studies ranged from low (0–3 score, 37 studies) to moderate (4–6 score, 6 studies). No study had a high risk of bias, six (13.9%) studies had moderate risk, and 37 (86.1%) had low risk (Table S2). Out of 43 studies, 34 were prospective cross-sectional, six were retrospective cross-sectional, two were prospective cohort, and one was a case-control study (Table 1).

### 3.2. Dengue Virus Outbreaks and Serotype Distribution

**Figure 2:**
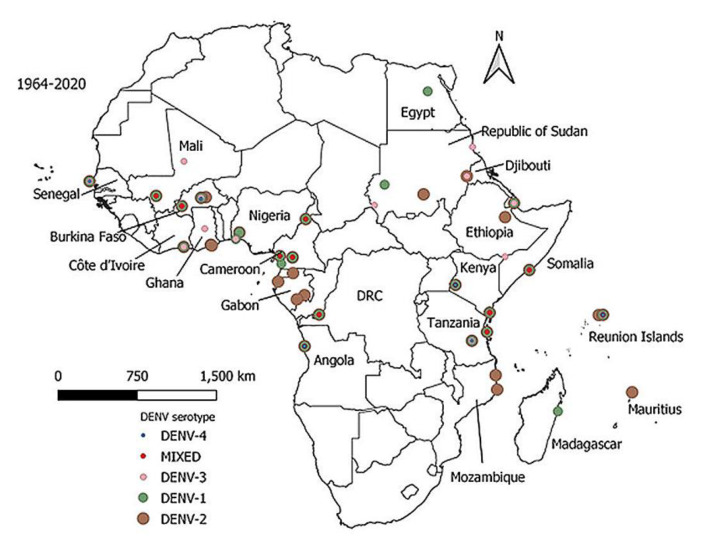
Distribution of dengue virus serotypes in Africa, 1964–2020. DENV-2 was the dominant serotype (brown), followed by DENV-1 (dark green), DENV-3 (light pink), Mixed serotypes (red) and DENV-4 (blue). The map was developed using Quantitative Geographic Information System (QGIS) open-source software version 3.16 available at https://www.qgis.org/en/site/forusers/download (accessed on 12 February 2021).

**Figure 3:**
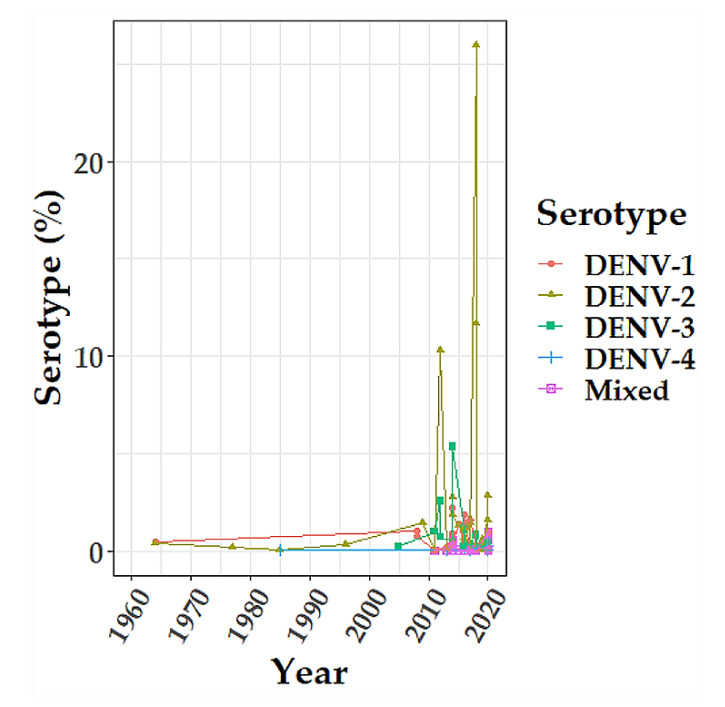
The longitudinal trend of dengue virus serotype proportion in Africa, 1964- 2020. The color codes represent DENV-1 (red), DENV-2 (dark khaki), DENV-3 (green), DENV-4 (blue), and Mixed serotype (pink). The graph was created using R software version 3.5.2 with a primary package ggplot2 at https://cran.r-project.org/bin/windows/base/old/3.5.2/ (accessed on 3 December 2020).

Since 1964, 45 dengue outbreaks were reported in 14 countries (Table 2). Most of the outbreaks occurred in East (17/45) and West (16/45) Africa. DENV-1 and DENV-2 were dominant serotypes in most of the outbreaks (Figure 2). During the past decade (2010–2020), there was an expansion of multiple DENV serotypes occurrence in Africa (Figure 3).

### 3.3. Dengue Prevalence

**Figure 4:**
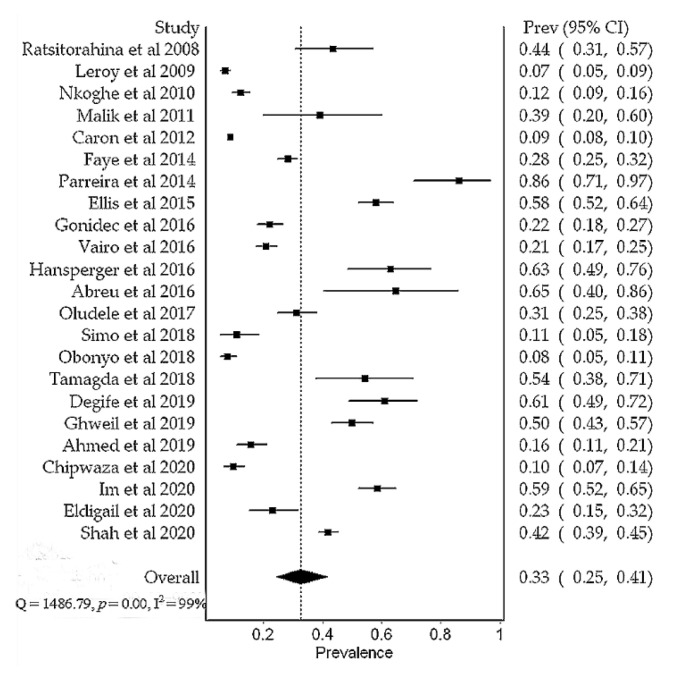
A meta-analysis of dengue virus prevalence in febrile patients during the outbreak periods.

**Figure 5:**
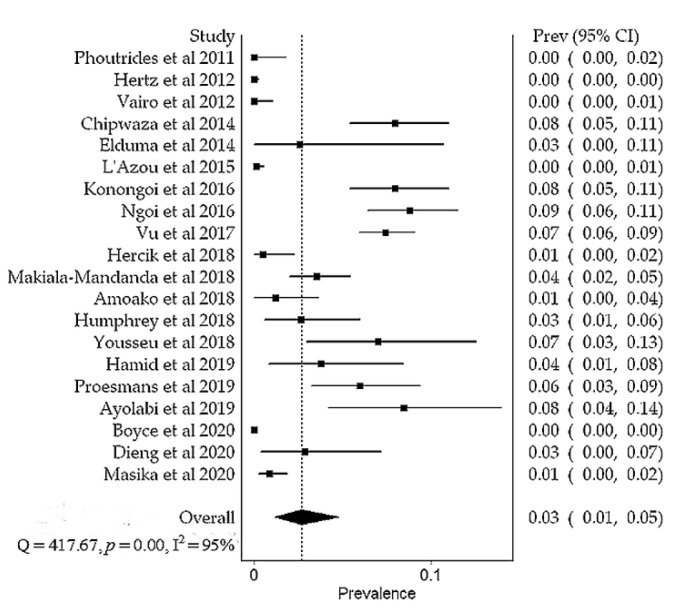
A meta-analysis of dengue virus prevalence in febrile patients during the non-outbreak periods.

**Figure 6:**
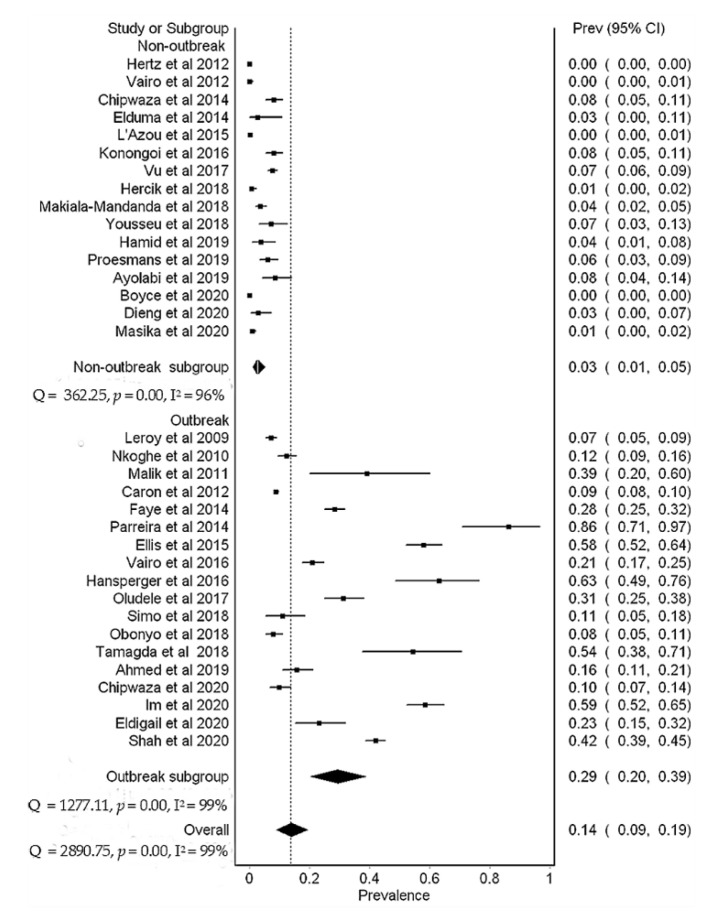
A subgroup meta-analysis of dengue virus prevalence in febrile patients based on prospective cross-sectional studies.

Overall, the prevalence of DENV in Africa was 14% (95% CI, 9–19%), N = 15,807). Substantial heterogeneity was found between studies during outbreak (I^2^ = 99%, *p* < 0.01) and non-outbreak (I^2^ = 95%, *p* < 0.01) periods (Figure 4 and Figure 5). Subgroup meta-analysis showed that the prevalence of DENV was 29% (95% CI, 20–39%, N = 8966) and 3% (95% CI, 1–5%, N = 6841) during outbreak and non-outbreak periods, respectively (Figure 6). Sensitivity analysis based on prospective cross-sectional studies (*n* = 34/43) showed that 33/34 studies had good precision on overall DENV prevalence (14% (95% CI, 9–20%). One study had relatively low precision (12% (95% CI, 8–18%) [23] (Table 3). Funnel plot asymmetry (Figure S1) and Egger’s regression test (*p* = 0.0022) indicated an evidence of publication bias in the outbreak studies. No evidence of publication bias (Figure S2) was detected in the non-outbreak studies (Egger’s regression test, *p* = 0.2633).

### 3.4. Severe Dengue and Co-Morbidities

Between 2011 and 2019, a total of 176 severe dengue cases were reported in six countries: Burkina Faso, Côte d’Ivoire, Djibouti, Republic of Sudan, Senegal, and Tanzania. The majority of cases were reported in the Republic of Sudan (126/176) and Burkina Faso (38/176). Malaria and dengue co-infections were the most prevalent (78%, 554/711), followed by dengue and chikungunya co-infections (16%, 114/711). Other co-morbidities of dengue were yellow fever, measles, pancreatitis, and hepatitis E (6%, 43/711).

### 3.5. Risk Factors

Evidence from 21 reports published between 2007 and 2020 showed that old age, lack of mosquito control, living in urban areas, climate change, and history of recent travel were the leading risk factors of dengue. Other risk factors were type of occupation, lack of education, low income, and known diabetes mellitus status (Table 4).

## 4. Discussion

This systematic review reports the distribution of outbreaks and the prevalence of dengue in Africa during the outbreak and non-outbreak periods. Our results show that dengue has been reported in 24 of 54 countries and has become endemic, with repeated outbreaks in most of them. Since 1960, all four DENV serotypes (DENV-1–4) caused epidemics in all African sub-regions, with DENV-1 and DENV-2 dominating. Laboratory confirmed outbreaks were reported in 13 African countries, with the East Africa region contributing over 50% of the epidemics. These observations support evidence previously documented [5,7]. After 2010, severe dengue cases have been increasingly reported in different countries, including Burkina Faso [79], Côte d’Ivoire [80], Djibouti [30], the Republic of Sudan [81], Senegal [22], and Tanzania [31]. The previous report shows that these countries have experienced continuous active DENV transmission in the past decade [5].

Our analysis revealed an increased occurrence of multiple DENV serotypes in Africa during the past decade (2010–2020) (Figure 3), with a greater proportion of serotypes reported in East and West Africa (Figure 2). Concurrent infections with multiple serotypes may pose a risk of severe dengue because lifelong immunity against primary infection by one serotype does not cross-protect subsequent infections by a different serotype. In secondary infection, antibody-dependent enhancement facilitates viral multiplication in the host cells, resulting in severe disease [82]. Expansion of multiple DENV serotypes in Africa may be caused by several factors. International travel of infected people from epidemic and endemic countries has been associated with the introduction of DENV-1 and DENV-3 serotypes in several African countries [83,84]. Spill-over of sylvatic DENV-2 strains from forest Aedes mosquitoes into a human transmission cycle possibly facilitates the spread into urban or new geographical areas with the potential to cause epidemics [85,86]. Further, increasing recognition of DENV as the cause of undifferentiated febrile syndromes [87], and the availability of more sensitive and specific molecular-based laboratory tests in the past decade, may have contributed to more detection and reporting of DENV serotypes [51,56,88].

Meta-analysis results show that the overall prevalence of dengue virus in Africa is 14%. This prevalence is relatively higher than the 7% reported in previous meta-analysis [36]. The discrepancy could be due to differences in a number of prevalence studies included in the meta-analysis. More studies included in this review possibly contributed to an increased number of dengue positive cases. In addition, our review included studies conducted during outbreaks, thus, large studies with a higher proportion of dengue positive cases were expected. During an outbreak, the prevalence of dengue virus was 29%. Our results agree with the 30% prevalence reported in the previous meta-analysis that included prospective cross-sectional studies conducted during epidemics or following recognized epidemics in the Republic of Sudan [89]. These observations indicate a high burden of dengue of up to 39% in febrile patients during an outbreak, highlighting the need for routine laboratory dengue diagnosis in tropical Africa.

Low dengue virus prevalence of 3% in febrile patients was found during the non-outbreak period (Figure 6). In comparison, our results were relatively lower than values reported in a previous meta-analysis by Simo et al. (2019) involving febrile patients from studies conducted during the non-outbreak period [36]. This difference could be due to the selection and epidemiological contexts of included studies. For instance, inclusion of studies conducted during ongoing epidemics or following epidemics are likely to contribute to a higher number of dengue positive cases [32]. As a result, the overall prevalence could have been overestimated. Despite the low prevalence observed during the non-outbreak period, a burden of up to 5% in febrile patients is still of a public health concern that needs appropriate interventions. Persistent occurrence of sporadic dengue cases may indicate endemicity, therefore, routine laboratory dengue diagnosis and enhanced mosquito surveillance could help to detect cases early and identify hotspots, respectively. Despite substantial variability (I^2^ = 98.89) between prospective cross-sectional studies, more than 90% of the studies had good precision on the overall dengue prevalence (Table 3) and low risk of methodological quality bias (Table S2). The presence of publication bias (Egger’s test, *p* = 0.0022) in the outbreak studies (Figure S1), could be due to small studies with non-significant results not being published.

Co-existing unrecognized co-morbidities can complicate dengue diagnosis and patient management. The findings from previous studies [90,91] show that co-morbidities increase the risk of severe disease and fatal outcomes among dengue patients. Our results show that in the past decade (2010–2020), malaria and dengue co-infections were the most prevalent, followed by dengue and chikungunya co-infection [24,31,40,43,53,56,79,92,93,94,95]. A similar occurrence pattern of malaria and dengue co-infection dominance followed by dengue and chikungunya co-infection was previously reported [96]. In Africa, co-morbidities of dengue are not usually diagnosed due to a lack of diagnostic capacity to differentiate dengue from other mosquito-borne acute febrile illnesses such as chikungunya, Zika, and yellow fever. The diseases develop similar non-specific clinical signs and can be co-transmitted with dengue [97]. These findings underscore the need to enhance differential diagnosis of non-malaria febrile illnesses in Africa.

Results from this review show that increasing age, lack of mosquito control, living in an urban area, climate change, and recent history of travel were the leading risk factors of dengue in Africa (Table 4). A high risk of contracting DENV in the old age group may be explained by continuous exposure to Flaviviruses [54,69,72]. The presence and abundance of Aedes mosquito vectors are known to increase risk of dengue exposure [98,99]. Evidence from some studies shows that people living in areas surrounding waste dump sites, opening windows at night, presence of stagnant water at home, households with indoor bathrooms, and living with open water containers were associated with a high risk of dengue [27,45]. Other potential risk factors included occupation type, lack of education, low income, and known diabetes mellitus status [48,77]. These findings disclose gaps in individual and environmental practices that could limit Aedes mosquito abundance and spread in African settings.

This review had some limitations. First, we could not establish a meta-analysis of DENV NS1 prevalence due to an inadequate number of studies reporting NS1 prevalence alone. Most studies had overlap data between NS1 and RT-PCR test. Second, a small number of studies (*n* < 10) limited subgroup meta-analysis of dengue prevalence based on setting (community versus healthcare facility), geographical sub-regions, and the design other than prospective cross-sectional. Third, it is possible that some individuals were asymptomatic and could not be detected in the included studies, thus, the number of dengue positive cases may be higher than reported in this review. Despite the limitations, we are confident that our findings provide useful information for clinical practice and public health policy decisions.

## 5. Conclusions

In conclusion, this review reveals a high burden of dengue infection and highlights an increased risk of severe disease in Africa due to the increasing circulation of multiple dengue virus serotypes. We advocate for the need of routine laboratory dengue diagnosis in Africa to facilitate early detection of cases, provision for appropriate patient care, identification of serotypes/genotypes, and outbreak preparedness. It is important to implement effective mosquito surveillance to identify hotspots, and control through the promotion of education on individual behaviours and environmental management practices that can limit the spread of dengue infection in Africa.

## Figures and Tables

**Table 1:** The characteristics of studies included in the systematic review and meta-analysis by country, region, design, and population.

s/n	Reference	Country	Region	Design	Population	Cases	Sample Size	Epidemiological Context	Risk of Bias
1	Ratsitorahina et al. [14]	Madagascar	East Africa	Retrospective cross-sectional	Dengue confirmed febrile	24	55	Outbreak	Low
2	Leroy et al. [15]	Gabon	Central Africa	Prospective cross-sectional	Dengue confirmed febrile	54	773	Outbreak	Low
3	Nkoghe et al. [16]	Gabon	Central Africa	Prospective cross-sectional	Dengue confirmed febrile	53	433	Outbreak	Low
4	Malik et al. [17]	Republic of Sudan	North Africa	Prospective cross-sectional	Dengue confirmed febrile	9	23	Outbreak	Low
5	Phoutrides et al. [18]	Mali	West Africa	Retrospective cross-sectional	Febrile patients	0	95	Non-outbreak	Moderate
6	Caron et al. [19]	Gabon	Central Africa	Prospective cross-sectional	Dengue confirmed febrile	376	4287	Outbreak	Low
7	Hertz et al. [20]	Tanzania	East Africa	Prospective cross-sectional	Febrile patients	0	700	Non-outbreak	Low
8	Vairo et al. [21]	Tanzania	East Africa	Prospective cross-sectional	Febrile patients	0	165	Non-outbreak	Low
9	Faye et al. [22]	Senegal	West Africa	Prospective cross-sectional	Dengue confirmed febrile	196	696	Outbreak	Low
10	Parreira et al. [23]	Angola	South Africa	Prospective cross-sectional	Dengue confirmed febrile	25	29	Outbreak	Low
11	Chipwaza et al. [24]	Tanzania	East Africa	Prospective cross-sectional	Dengue confirmed febrile	29	364	Non-outbreak	Low
12	Elduma et al. [25]	Republic of Sudan	North Africa	Prospective cross-sectional	Dengue confirmed febrile	1	39	Non-outbreak	Moderate
13	L’Azou et al. [26]	Côte d’Ivoire	West Africa	Prospective cross-sectional	Febrile patients	1	796	Non-outbreak	Low
14	Ellis et al. [27]	Kenya	East Africa	Prospective cross-sectional	Dengue confirmed febrile	155	267	Outbreak	Low
15	Konongoi et al. [28]	Kenya	East Africa	Prospective cross-sectional	Dengue confirmed febrile	29	364	Non-outbreak	Low
16	Ngoi et al. [29]	Kenya	East Africa	Prospective cohort	Dengue confirmed febrile	43	489	Non-outbreak	Moderate
17	Gonidec et al. [30]	Djibouti	East Africa	Retrospective cross-sectional	Dengue confirmed febrile	78	354	Outbreak	Low
18	Vairo et al. [31]	Tanzania	East Africa	Prospective cross-sectional	Dengue confirmed febrile	101	483	Outbreak	Low
19	Hansperger et al. [32]	Angola	South Africa	Prospective cross-sectional	Dengue confirmed febrile	29	46	Outbreak	Moderate
20	Abreu et al. [33]	Angola	South Africa	Retrospective cross-sectional	Dengue confirmed febrile	11	17	Outbreak	Moderate
21	Vu et al. [34]	Kenya	East Africa	Prospective cross-sectional	Dengue confirmed febrile	82	1104	Non-outbreak	Moderate
22	Oludele et al. [35]	Mozambique	South Africa	Prospective cross-sectional	Dengue confirmed febrile	60	192	Outbreak	Low
23	Simo et al. [36]	Cameroon	West Africa	Prospective cross-sectional	Dengue confirmed febrile	10	91	Outbreak	Low
24	Hercik et al. [37]	Tanzania	East Africa	Prospective cross-sectional	Dengue confirmed febrile	1	191	Non-outbreak	Low
25	Obonyo et al. [38]	Kenya	East Africa	Prospective cross-sectional	Dengue confirmed febrile	30	381	Outbreak	Low
26	Makiala et al. [39]	DRC *	Central Africa	Prospective cross-sectional	Dengue confirmed febrile	16	453	Non-outbreak	Low
27	Amoako et al. [40]	Ghana	West Africa	Retrospective cross-sectional	Dengue confirmed febrile	2	166	Non-outbreak	Low
28	Humphrey et al. [41]	Ghana	West Africa	Retrospective cross-sectional	Dengue confirmed febrile	4	150	Non-outbreak	Low
29	Tarnagda et al. [42]	Burkina Faso	West Africa	Prospective cross-sectional	Dengue confirmed febrile	19	35	Outbreak	Low
30	Yousseu et al. [43]	Cameroon	West Africa	Prospective cross-sectional	Dengue confirmed febrile	8	114	Non-outbreak	Low
31	Hamid et al. [44]	Republic of Sudan	North Africa	Prospective cross-sectional	Dengue confirmed febrile	4	106	Non-outbreak	Low
32	Degife et al. [45]	Ethiopia	East Africa	case control	Dengue suspects	42	69	Outbreak	Low
33	Ghweil et al. [46]	Egypt	North Africa	Prospective cohort	Dengue confirmed febrile	100	200	Outbreak	Low
34	Ahmed et al. [47]	Republic of Sudan	North Africa	Prospective cross-sectional	Dengue confirmed febrile	32	204	Outbreak	Low
35	Proesmans et al. [48]	DRC *	Central Africa	Prospective cross-sectional	Dengue confirmed febrile	14	235	Non-outbreak	Low
36	Ayolabi et al. [49]	Nigeria	West Africa	Prospective cross-sectional	Dengue confirmed febrile	11	130	Non-outbreak	Low
37	Boyce et al. [50]	Uganda	East Africa	Prospective cross-sectional	Febrile patients	0	1416	Non-outbreak	Low
38	Chipwaza et al. [51]	Tanzania	East Africa	Prospective cross-sectional	Dengue confirmed febrile	29	294	Outbreak	Low
39	Dieng et al. [52]	Senegal	West Africa	Prospective cross-sectional	Dengue confirmed febrile	3	104	Non-outbreak	Low
40	Im et al. [53]	Burkina Faso	West Africa	Prospective cross-sectional	Dengue confirmed febrile	141	241	Outbreak	Low
41	Eldigail et al. [54]	Republic of Sudan	North Africa	Prospective cross-sectional	Dengue confirmed febrile	23	100	Outbreak	Low
42	Masika et al. [55]	Kenya	East Africa	Prospective cross-sectional	Dengue confirmed febrile	5	560	Non-outbreak	Low
43	Shah et al. [56]	Kenya	East Africa	Prospective cross-sectional	Dengue confirmed febrile	361	862	Non-outbreak	Low

* DRC = Democratic Republic of Congo.

**Table 2:** Dengue virus outbreaks in Africa by year, country, sub-region, and serotype, 1964–2020.

S/n	Year of Outbreak	Country	Sub-Region	Serotype	Reference
1	1964	Nigeria	West Africa	DENV-1/2	[57]
2	1977	Seychelles	East Africa	DENV-2	[58]
3	1985	Senegal	West Africa	DENV-2/4	[59]
4	1985	Somalia	East Africa	DENV-2	[60]
5	2005	Republic of Sudan	North Africa	DENV-3	[17]
6	2007	Gabon	Central Africa	DENV-2	[15]
7	2008	Mali	West Africa	DENV-3	[61] **
8	2008	Madagascar	East Africa	DENV-1	[14]
9	2009	Cape Verde	West Africa	NR *	[62] ***
10	2009	Mauritius	East Africa	DENV-2	[63]
11	2009	Senegal	West Africa	DENV-3	[22]
12	2010	Gabon	Central Africa	NR	[61]
13	2010	Côte d’Ivoire	West Africa	NR	[61]
14	2011	Republic of Sudan	North Africa	DENV-3	[17]
15	2012	Republic of Sudan	North Africa	DENV-3	[64]
16	2013	Angola	Southern Africa	NR	[61]
17	2013	Ethiopia	East Africa	DENV-2	[65]
18	2013	Kenya	East Africa	DENV-1/2	[27]
19	2014	Tanzania	East Africa	DENV-2	[31]
20	2015	Egypt	North Africa	DENV-1	[62]
21	2015	Ethiopia	East Africa	NR	[45]
22	2015	Republic of Sudan	North Africa	DENV-1/3	[47]
23	2016	Burkina Faso	West Africa	DENV-1/3	[53]
24	2016	Reunion Islands	East Africa	DENV-1–4	[66]
25	2016	Seychelles	East Africa	NR	[61]
26	2016	Burkina Faso	West Africa	DENV-2/3	[42]
27	2016	Angola	Southern Africa	DENV-3/4	[23]
28	2016	Djibouti	East Africa	DENV-1–3	[30]
29	2016	Mozambique	Southern Africa	DENV-2	[35]
30	2017	Burkina Faso	West Africa	DENV-1–3	[62]
31	2017	Côte d’Ivoire	West Africa	DENV-1–3	[62]
32	2017	Kenya	East Africa	NR	[62]
33	2017	Côte d’Ivoire	West Africa	NR	[61]
34	2017	Burkina Faso	West Africa	NR	[61]
35	2017	Senegal	West Africa	NR	[61]
36	2017	Reunion Islands	East Africa	DENV-1/2/4	[66]
37	2018	Tanzania	East Africa	DENV-1–4	[51]
38	2018	Senegal	West Africa	NR	[61]
39	2018	Mauritania	North Africa	NR	[61]
40	2018	Reunion Islands	East Africa	DENV-2	[66]
41	2019	Reunion Islands	East Africa	NR	[61]
42	2019	Côte d’Ivoire	West Africa	DENV-1/3	[61]
43	2019	Tanzania	East Africa	DENV-1	[61]
44	2019	Republic of Sudan	North Africa	DENV-3	[54]
45	2020	Mauritania	North Africa	NR	[61]

* NR = serotype not reported; ** ProMED-mail source: https://promedmail.org/ (accessed on 3 December 2020); *** WHO source: https://www.who.int/csr/don/en/ (accessed on 3 December 2020).

**Table 3:** Sensitivity analysis of dengue virus prevalence based on prospective cross-sectional studies.

Excluded Study	Pooled Prevalence (95% CI)	I^2^ (95% CI)	*p*-Value
Outbreak Studies (*n* = 18)			
Leroy et al., 2009	0.14 (0.09, 0.20)	98.89 (98.78, 98.74)	<0.01
Nkoghe et al., 2010	0.14 (0.09, 0.20)	98.89 (98.74, 99.03)	<0.01
Malik et al., 2011	0.13 (0.08, 019)	98.89 (98.73, 99.02)	<0.01
Caron et al., 2012	0.14 (0.08, 0.21)	98.89 (98.74, 99.03)	<0.01
Faye et al., 2014	0.13 (0.08, 0.19)	98.89 (98.64, 98.96)	<0.01
Parreira et al., 2014	0.12 (0.08, 0.18)	98.89 (98.74, 99.03)	<0.01
Ellis et al., 2015	0.13 (0.08, 0.18)	98.89 (98.54, 98.90)	<0.01
Vairo et al., 2016	0.13 (0.09, 0.19)	98.89 (98.71, 99.01)	<0.01
Hansperger et al., 2016	0.13 (0.08, 0.18)	98.89 (98.71, 99.00)	<0.01
Oludele et al., 2017	0.13 (0.08, 0.19)	98.89 (98.71, 99.01)	<0.01
Simo et al., 2018	0.14 (0.09, 0.20)	98.89 (98.74, 99.03)	<0.01
Obonyo et al., 2018	0.14 (0.09, 0.20)	98.89 (98.74, 99.03)	<0.01
Tamagda et al., 2018	0.13 (0.08, 0.18)	98.89 (98.72, 99.01)	<0.01
Ahmed et al., 2019	0.14 (0.09, 0.19)	98.89 (98.74, 99.02)	<0.01
Chipwaza et al., 2020	0.14 (0.09, 0.20)	98.89 (98.74, 99.03)	<0.01
Im et al., 2020	0.13 (0.08, 0.18)	98.89 (98.57, 98.91)	<0.01
Eldigail et al., 2020	0.13 (0.09, 0.19)	98.89 (98.73, 99.02)	<0.01
Shah et al., 2020	0.13 (0.09, 0.18)	98.89 (98.39, 98.79)	<0.01
Non-Outbreak Studies (*n* = 16)			
Hertz et al., 2012	0.14 (0.10,0.20)	98.89 (98.63, 98.95)	<0.01
Vairo et al., 2012	0.14 (0.09, 0.20)	98.89 (98.72, 99.01)	<0.01
Chipwaza et al., 2014	0.14 (0.09, 0.20)	98.89 (98.74, 99.03)	<0.01
Elduma et al., 2014	0.14 (0.09, 0.20)	98.89 (98.74, 99.03)	<0.01
L’Azou et al., 2015	0.14 (0.09, 0.20)	98.89 (98.63, 98.95)	<0.01
Konongoi et al., 2016	0.14 (0.09, 0.20)	98.89 (98.74, 99.03)	<0.01
Vu et al., 2017	0.14 (0.09, 020)	98.89 (98.74, 99.03)	<0.01
Hercik et al., 2018	0.14 (0.09, 0.20)	98.89 (98.72, 99.02)	<0.01
Makiala-Mandanda et al., 2018	0.14 (0.09, 0.20)	98.89 (98.73, 99.02)	<0.01
Yousseu et al., 2018	0.14 (0.09, 0.20)	98.89 (98.74, 99.03)	<0.01
Hamid et al., 2019	0.14 (0.09, 0.20)	98.89 (98.74, 99.03)	<0.01
Proesmans et al., 2019	0.14 (0.09, 0.20)	98.89 (98.74, 99.03)	<0.01
Ayolabi et al., 2019	0.14 (0.09, 0.20)	98.89 (98.74, 99.03)	<0.01
Boyce et al., 2020	0.14 (0.10, 0.20)	98.89 (98.46, 98.83)	<0.01
Dieng et al., 2020	0.14 (0.09, 0.20)	98.89 (98.74, 99.03)	<0.01
Masika et al., 2020	0.14 (0.09, 020)	98.89 (98.70, 99.00)	<0.01

**Table 4:** Potential risk factors of dengue virus infection in Africa (*n* = 21 studies).

Factor	No. Studies	Risk Category	Rank	Reference
Increasing old age	6	Socio-demographic	1	[21,48,54,67,68,69]
Lack of mosquito control	6	Environmental	1	[27,45,67,70,71,72]
Urban residence	4	Socio-demographic	2	[28,54,69,73]
Climate change	3	Ecological	3	[19,29,74]
History of recent travel	3	Environmental	3	[27,48,75]
Occupation type	2	Socio-demographic	4	[68,76]
Lack of education	2	Socio-demographic	4	[68,77]
Low income	1	Socio-demographic	5	[54]
Known diabetes mellitus	1	Health	5	[78]

## Supplementary Materials

The following are available online at https://www.mdpi.com/1999-4915/13/4/536/s1, Table S1: Quality assessment checklist, Table S2: Risk of bias scores, Figure S1: Funnel plot for the outbreak studies, Figure S2: Funnel plot for the non-outbreak studies.

## Appendix A

**Table A1:** Literature search strategy for studies included in the systematic review and meta-analysis. Search date: October to December, 2020.

Key Search Terms:
(Dengue) [Title] AND (Africa = list of countries) [Title]
(Dengue) [Title] AND (Africa = list of countries) [Title] AND “Outbreak” [Key words]
(Dengue) [Title] AND (Africa = list of countries) [Title] AND “Prevalence” [Key words]
(Dengue) [Title] AND (Africa = list of countries) [Title] AND “Co-morbidies” OR “Co-infections” [Key words]
(Dengue) [Title] AND (Africa = list of countries) [Title] AND “Risk factors” [Key words]

(African countries = “Algeria OR Angola OR Benin OR Botswana OR Burkina Faso OR Burundi OR Cameroon OR Cape Verde OR Central African Republic OR Chad OR Comoros OR Congo (Brazzaville) OR Congo (Democratic Republic) OR Côte d’Ivoire OR Djibouti OR Egypt OR Equatorial Guinea OR Eritrea OR Ethiopia OR Gabon OR Gambia OR Ghana OR Guinea OR Guinea-Bissau OR Kenya OR Lesotho OR Liberia OR Libya OR Madagascar OR Malawi OR Mali OR Mauritania OR Mauritius OR Morocco OR Mozambique OR Namibia OR Niger OR Nigeria OR Reunion OR Rwanda OR Sao Tome and Principe OR Senegal OR Seychelles OR Sierra Leone OR Somalia OR South Africa OR Sudan OR South Sudan OR Swaziland OR Tanzania OR Togo OR Tunisia OR Uganda OR Western Sahara OR Zambia OR Zimbabwe OR Africa OR East Africa OR West Africa OR Southern Africa OR Central Africa OR North Africa”). Publication year limit: 1960–2020 (inclusive).
