# Supplementary material for: Dengue Virus Infection and Associated Risk Factors in Africa: A Systematic Review and Meta-Analysis
Source: Viruses. 2021 Mar 24;13(4):536. doi: 10.3390/v13040536 (PMC8063827; doi:10.3390/v13040536)
Supplement: Supplementary file 1 [file viruses-13-00536-s001.zip › Table S2_Risk of bias scores.pdf]

Table S2: Risk of bias scores for individual studies included in the review

S/n	Author	Year	Study Design	Total score	Score level	Risk of bias
1	Ratsitorahina et al	2008	Retrospective cross-sectional	1	0-3	Low
2	Leroy et al	2009	Prospective cross-sectional	3	0-3	Low
3	Nkoghe et al	2010	Prospective cross-sectional	2	0-3	Low
4	Becquart et al	2010	Prospective cross-sectional	1	0-3	Low
5	Malik et al	2011	Prospective cross-sectional	2	0-3	Low
6	Phoutrides et al	2011	Retrospective cross-sectional	4	4-6	Moderate
7	Caron et al	2012	Prospective cross-sectional	2	0-3	Low
8	Hertz et al	2012	Prospective cross-sectional	1	0-3	Low
9	Vairo et al.	2012	Prospective cross-sectional	2	0-3	Low
10	Faye et al	2014	Prospective cross-sectional	3	0-3	Low
11	Parreira et al	2014	Prospective cross-sectional	2	0-3	Low
12	Chipwaza et al	2014	Prospective cross-sectional	2	0-3	Low
13	Elduma et al	2014	Prospective cross-sectional	4	4-6	Moderate
14	Ellis et al	2015	Prospective cross-sectional	1	0-3	Low
15	L'Azou et al	2015	Prospective cross-sectional	2	0-3	Low
16	Konongoi et al	2016	Prospective cross-sectional	3	0-3	Low
17	Ngoi et al	2016	Prospective cohort	2	0-3	Moderate
18	Gonidec et al	2016	Retrospective cross-sectional	3	0-3	Low
19	Vairo et al	2016	Prospective cross-sectional	2	0-3	Low
20	Hansperger et al	2016	Prospective cross-sectional	4	4-6	Moderate
21	Abreu et al	2016	Retrospective cross-sectional	4	4-6	Moderate
22	Vu et al	2017	Prospective cross-sectional	4	4-6	Moderate
23	Oludele et al	2017	Prospective cross-sectional	3	0-3	Low
24	Simo et al	2018	Prospective cross-sectional	3	0-3	Low
25	Obonyo et al	2018	Prospective cross-sectional	1	0-3	Low
26	Hercik et al	2018	Prospective cross-sectional	3	0-3	Low
27	Makiala-Mandanda et al	2018	Prospective cross-sectional	1	0-3	Low
28	Amoako et al	2018	Retrospective cross-sectional	1	0-3	Low
29	Humphrey et al	2018	Retrospective cross-sectional	1	0-3	Low
30	Tamagda et al	2018	Prospective cross-sectional	3	0-3	Low
31	Yousseu et al	2018	Prospective cross-sectional	1	0-3	Low
32	Hamid et al	2019	Prospective cross-sectional	2	0-3	Low
33	Degife et al	2019	case control	1	0-3	Low
34	Ghweil et al	2019	Prospective cohort	3	0-3	Low
35	Ahmed et al	2019	Prospective cross-sectional	2	0-3	Low
36	Proesmans et al	2019	Prospective cross-sectional	3	0-3	Low
37	Ayolabi et al	2019	Prospective cross-sectional	2	0-3	Low
38	Boyce et al	2020	Prospective cross-sectional	2	0-3	Low
39	Dieng et al	2020	Prospective cross-sectional	3	0-3	Low
40	Im et al	2020	Prospective cross-sectional	2	0-3	Low
41	Eldigail et al	2020	Prospective cross-sectional	3	0-3	Low
42	Masika et al	2020	Prospective cross-sectional	2	0-3	Low
43	Shah et al	2020	Prospective cross-sectional	1	0-3	Low
