# Supplementary figures and images for: Dengue Virus Infection and Associated Risk Factors in Africa: A Systematic Review and Meta-Analysis
Source: Viruses. 2021 Mar 24;13(4):536. doi: 10.3390/v13040536 (PMC8063827; doi:10.3390/v13040536)

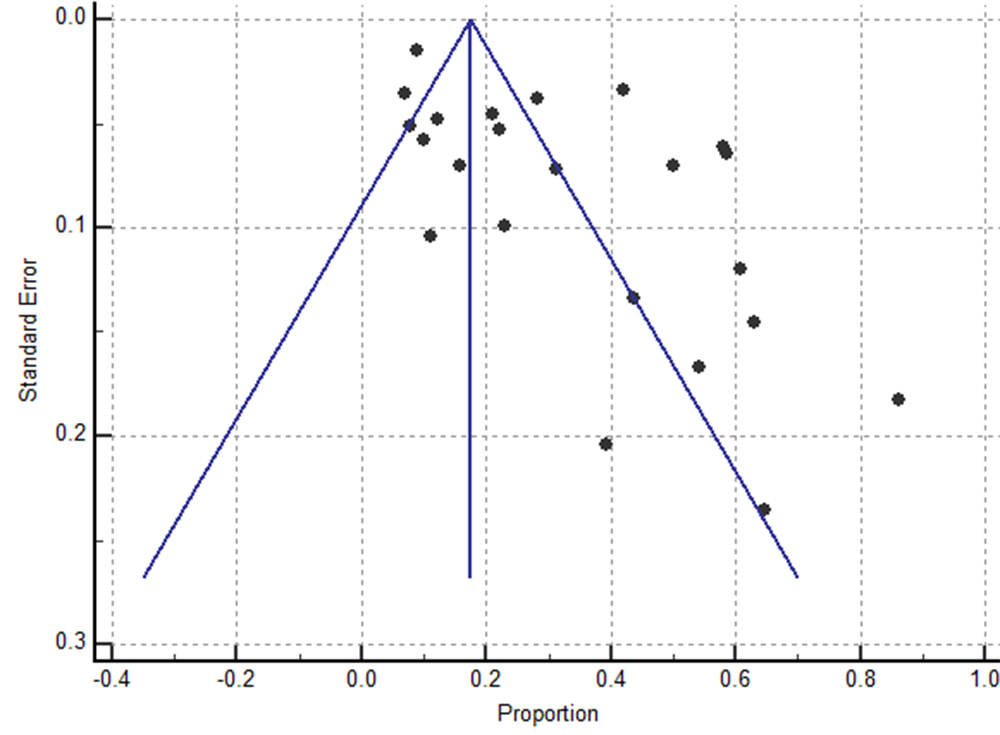

Supplement: Supplementary file 1 [file viruses-13-00536-s001.zip › Figure S1 Funnel plot_outbreak studies.png]

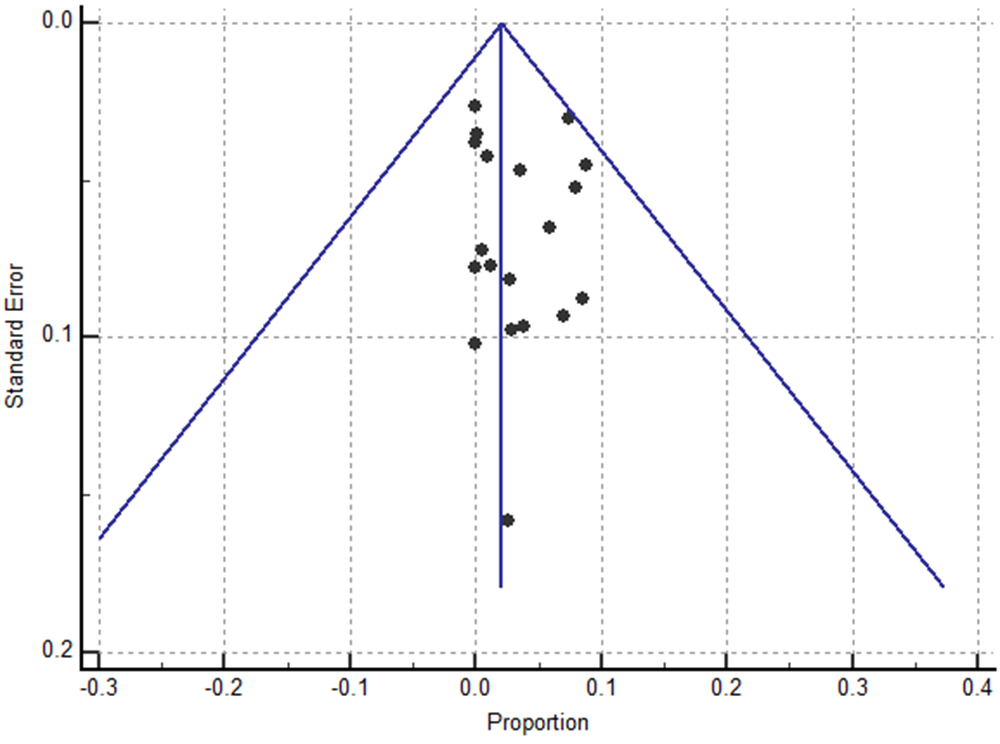

Supplement: Supplementary file 1 [file viruses-13-00536-s001.zip › Figure S2 Funnel plot_non-outbreak studies.png]
